# Neurocognitive Outcomes After Critical Illness: Post-ICU Syndrome in Sepsis Survivors

**DOI:** 10.14789/ejmj.JMJ25-0032-OA

**Published:** 2025-12-10

**Authors:** ANURAJ KODOTH VEETIL KOLLAMPADY, MUDASSAR IJAZ, ISSAM AHMED SAEED, HAROON JAVED, AHMAD HASAN, NUMAN AKRAM, MAHRUKH NAZEER, ABDULLAH IMTIAZ, MUHAMMAD WASEEM ATHER, SHAUMILE HASAN KHAN, ZAINAB SALAHUDDIN, SHER BANO

**Affiliations:** 1 Department of Cardiology, Frimley Park Hospital, Camberley, UK; 1 Department of Cardiology, Frimley Park Hospital, Camberley, UK; 2 Department of Psychiatry, Gulab Devi Hospital, Lahore, Pakistan; 2 Department of Psychiatry, Gulab Devi Hospital, Lahore, Pakistan; 3 Department of Dermatology, Gujranwala Medical College Teaching Hospital, Gujranwala, Pakistan; 3 Department of Dermatology, Gujranwala Medical College Teaching Hospital, Gujranwala, Pakistan; 4 Department of Internal Medicine, Shifa International Hospital, Islamabad, Pakistan; 4 Department of Internal Medicine, Shifa International Hospital, Islamabad, Pakistan; 5 Department of Medicine, Assiut University Faculty of Medicine, Assiut, Egypt; 5 Department of Medicine, Assiut University Faculty of Medicine, Assiut, Egypt; 6 Department of Internal Medicine, Services Institute of Medical Sciences, Lahore, Pakistan; 6 Department of Internal Medicine, Services Institute of Medical Sciences, Lahore, Pakistan; 7 Department of General Medicine, Abwa Medical College, Khurrianwala, Pakistan; 7 Department of General Medicine, Abwa Medical College, Khurrianwala, Pakistan; 8 Department of Medicine, Wah Medical College, Wah Cantt, Pakistan; 8 Department of Medicine, Wah Medical College, Wah Cantt, Pakistan; 9 Department of Medicine, Qiqihar Medical University, Qiqihar, China; 9 Department of Medicine, Qiqihar Medical University, Qiqihar, China; 10 Department of Medicine, Fatima Memorial Hospital, Lahore, Pakistan; 10 Department of Medicine, Fatima Memorial Hospital, Lahore, Pakistan; 11 Department of Clinical Psychology, SZABIST University, Islamabad, Pakistan; 11 Department of Clinical Psychology, SZABIST University, Islamabad, Pakistan; 12 Department of Clinical Psychology, Shifa Tameer-e-Millat University, Islamabad, Pakistan; 12 Department of Clinical Psychology, Shifa Tameer-e-Millat University, Islamabad, Pakistan

**Keywords:** sepsis survivors, post-ICU syndrome, cognitive impairment, anxiety, quality of life

## Abstract

**Objectives:**

To measure neurocognitive abilities, psychological well-being, and quality of life (QOL) in survivors of adult sepsis post-intensive care unit (ICU) discharge in Pakistan and to establish clinical and demographic predictors of post-ICU outcomes.

**Design:**

A cross-sectional study at tertiary care hospitals in Islamabad, Pakistan.

**Methods:**

Structured questionnaires were used to evaluate 500 adult survivors of sepsis, 4-8 weeks after ICU discharge. Mini-Mental State Examination (MMSE) was used to measure cognitive functioning, Hospital Anxiety and Depression Scale (HADS) to measure psychological symptoms, and the Short Form-36 (SF-36) to measure QOL. Hospital records provided demographic and clinical information. Statistical procedures involved chi-square tests, Mann-Whitney U tests, Kruskal-Wallis tests, Spearman correlations, and multiple regression tests.

**Results:**

Among 500 participants, 284 (57%) were cognitively impaired (MMSE < 24), 300 (60%) reported borderline-to-clinical anxiety, and 250 (50%) were depressed. The QOL was low, with the mean SF-36 score being 35/100 (SD ± 12). The longer the ICU stay, the poorer the cognition, the more anxiety, and the lower the quality of life (p < 0.001). Poorer outcomes were also predicted by older age (> 60 years, n = 150; 30%) and female sex (n = 180; 36%).

**Conclusion:**

In Pakistan, cognitive impairment, psychological distress, and poor quality of life among sepsis survivors are high within weeks of ICU discharge. Longer ICU stay, comorbidities, female sex, and older age predict poorer outcomes. Early follow-up and organised rehabilitation measures are acutely required to enhance survivorship.

## Introduction

Post-critical illness recovery has become a significant health concern in the global world, and survivors are commonly reported to have long-term physical and psychological problems, including fatigue, weakness, depression, anxiety, and mental focus impairment^1)^. Recent research indicates that long-term neurocognitive impairments are common among survivors of critical illness and can continue to manifest over many years and have a deleterious impact on QOL, everyday functioning, and return to work^2)^. Advances in intensive care have raised the survival rates but have also resulted in an increasing number of long-term critically ill patients, often unable to breathe independently, and with high death rates and no specific treatment other than supportive measures^3)^.

Sepsis, the most frequent cause of ICU admission, has shown an improvement in short-term survival. Still, most survivors develop long-term complications such as immune dysfunction, frequent infections, poor quality of life, cognitive and psychological impairments, and a high rate of rehospitalisation^4, 5)^. Sepsis-associated encephalopathy is one of the most common complications of sepsis; this is a neuroinflammatory and hemodynamic injury that causes acute changes in mental status and may lead to long-term cognitive flattening, but is challenging to diagnose and treat^6)^. Severe sepsis is prevalent in about 30% of early ICU admissions, which often results in respiratory failure, and is a significant burden to critical care services^7)^.

Sepsis survivors exhibit psychiatric disorders, including anxiety, depression, and PTSD, as late as 9 months after ICU, and these are frequently linked to a slow physical recovery. Quality of life deteriorates significantly during ICU stay. It partially improves six months later; however, physical functioning, role limitations, and general health remain lower than before the ICU stay^8, 9)^. Predisposing factors to psychiatric morbidity are older age, pre- existing cognitive impairment, and some ICU treatment agents like dobutamine and haloperidol^10)^.

The cognitive and psychological consequences of sepsis survivors following ICU discharge are understudied in Pakistan, with local healthcare determinants, follow-up treatment, and social support potentially affecting recovery. The study aims to evaluate the neurocognitive functionality, psychological well- being, and QOL of adult sepsis survivors in Pakistani hospitals after discharge from the ICU, discovering the patterns and the severity of disparities and examining clinical factors during critical illness that may influence the outcome after ICU, to inform clinical strategies that could support the outcomes after ICU in the local setting.

## Materials and Methods

### Study design and setting

It was a cross-sectional study conducted on adult survivors of sepsis who had follow-up visits 4-8 weeks following ICU discharge in tertiary care hospitals located in Islamabad, Pakistan. Participants were recruited in an orderly manner at outpatient medical clinics at scheduled follow-up appointments. Structured interviewers completed tests to gather data in a quiet clinical environment, and pertinent clinical data were acquired via hospital records.

### Inclusion and exclusion criteria

The adults aged 18 years and above who had received an official diagnosis of sepsis upon admission to the ICU and then discharge were included, provided that they attended regular follow-up visits 4-8 weeks after ICU discharge and were stable enough to be assessed.

Patients who had a known history of significant neurocognitive disorders (e.g., dementia), acute delirium at the time of evaluation, severe sensory or communication deficits that restricted involvement, or who could not give informed consent were excluded.

### Sampling technique and sample size

A convenience sampling method was used, in which all the eligible sepsis survivors who attended regular follow-up between 4 and 8 weeks after being released from the ICU were invited to participate. The sample size was estimated by applying the formula of the World Health Organisation (WHO) using a confidence interval of 95%, a margin of error of 5%, and a presumed population proportion of 0.5 with the aim of maximising variability. A sample size of 384 was necessary to satisfy the study, but to increase the precision of the study and control non-response or incomplete data, a sample of 500 respondents was recruited.

### Data collection and procedure

Data collection was done using the structured questionnaire that included demographic, clinical, and standardised assessment parts. Age, sex, marital status, education, occupation, comorbidities, and ICU-related factors, including length of stay, were collected as demographic and clinical data and were subsequently examined against outcome variables. Neurocognitive functioning was assessed through the Mini-Mental State Examination (MMSE), a 30-item screening instrument evaluating orientation, attention, memory, language, and visuospatial skills, with a score range of 0 to 30 (the lower the score, the more impaired the individual is). Test-retest reliability of MMSE is high (r = 0.80 to 0.95) and has been published by Folstein et al. (1975), Psychological Assessment Resources, Florida, USA^11)^. The Hospital Anxiety and Depression Scale (HADS) by Zigmond and Snaith (1983) was used to assess psychological well-being. The instrument is comprised of 14 items and has two subscales (seven anxiety and seven depression). The scale ranges between 0 and 3, with subscale scores ranging between 0 and 21, with higher scores indicating a greater degree of symptom severity. The reported Cronbach alpha is 0.80 in anxiety and 0.76 in depression^12)^. The 36-item Short Form Health Survey (SF-36) measured the QOL with respect to health, and it was developed as a subset of the Medical Outcomes Study by Ware and Sherbourne (1992; RAND Corporation, Santa Monica, CA, USA). The SF-36 has eight health domains: physical functioning, role limitations by physical and emotional issues, vitality, emotional well-being, social functioning, pain, and general perceptions of health. Domain scores have a range of 0 to 100, and the higher the score, the better the health. Most domains have an internal consistency of over 0.80^13)^.

### Ethical considerations

The Institutional Review Board of Lumina Research Foundation (IRB-2025-0132) granted ethical approval. Informed consent was obtained by a written form after informing the participants about the study purpose, procedures, risks, and benefits using plain language. Participation was voluntary, and it was promised that declining to participate would have no impact on medical care. The research team received the codes of identification and limited access to the data, which ensured the confidentiality of the research, and no personal identifiers were reported. The research was developed in line with the ethical principles outlined in the Declaration of Helsinki, and those participants who revealed significant psychological distress or impaired cognition received corresponding clinical assessment.

### Statistical analysis

Data analyses were performed in IBM SPSS Statistics version 26 (IBM Corp.). The demographic characteristics and distributions of outcome variables were summarised using descriptive statistics (frequencies and percentages). Rank-order correlations by Spearman were used to test the relationship between ICU length of stay and post-ICU outcomes. Tests of group differences were performed based on nonparametric tests, such as the Mann-Whitney U test to compare the genders and the Kruskal-Wallis test to compare the ages. Multiple linear regression analyses were conducted to determine predictors of cognitive, psychological, and quality of life outcomes. Lastly, associations between categorical variables, such as age group and cognitive impairment, ICU length of stay, and gender, were evaluated by chi-square tests of independence. All tests were at a level of p 0.05 significance.

## Results

### Baseline characteristics

Table 1 presents the demographic characteristics of the respondents (N = 500). Those older than 60 years (n = 150, 30%) and 50-59 years (n = 125, 25%) constituted the majority, with most of them being male (n = 320, 64%). In terms of marital status, 180 (36%) were married, 148 (30%) were widowed, and 124 (25%) were single. The general level of education was low, with 184 (37%) having primary education and just 50 (10%) having intermediate or higher education. The largest employment groups (n = 174, 35% and n = 164, 33% respectively) were homemakers and students, and almost half of the population resided in semi-urban regions (n = 231, 46%). Most of them were former smokers (n = 264, 53%) and current smokers (n = 155, 31%). Lung disease (n = 168, 34%) and hypertension (n = 162, 32%) were the most common medical conditions, and 22 (4%) reported a history of depression or anxiety before ICU admission. Most participants stayed in ICU between 3 and 6 days (n = 201, 40% and 7-13 days (n = 164, 33%).

**Table 1:** Baseline characteristics of study participants (N = 500)

Variable	f	%		Variable	f	%
Age				Employed	64	13
18-29 years	50	10		Homemaker	174	35
30-39 years	75	15		Unemployed	17	3
40-49 years	100	20		Retired	81	16
50-59 years	125	25		Residence area		
60 years and above	150	30		Urban	132	26
Gender				Semi urban	231	46
Male	320	64		Rural	137	27
Female	180	36		Smoking use		
Marital status				Never smoked	81	16
Single	124	25		Former smoker	264	53
Married	180	36		Current smoker	155	31
Widowed	148	30		Do you have any of the following medical conditions?		
Divorced/separated	48	10		Diabetes	64	13
Educational level				Hypertension	162	32
No schooling	111	22		Lung disease (COPD, asthma)	168	34
Primary	184	37		Heart disease (stroke, MI, HF)	78	16
Middle	129	26		Depression/Anxiety before ICU	22	4
Matric/SSC	26	5		None	6	1
Intermediate	15	3		ICU length of stay		
Bachelor's degree	13	3		< 3 days	87	17
Master's or above	22	4		3-6 days	201	40
Employment				7-13 days	164	33
Student	164	33		≥14 days	48	10

f = frequency, % = percentage; Values are presented as N (%), N = 500.

### Cognition (MMSE)

**Figure 1:**
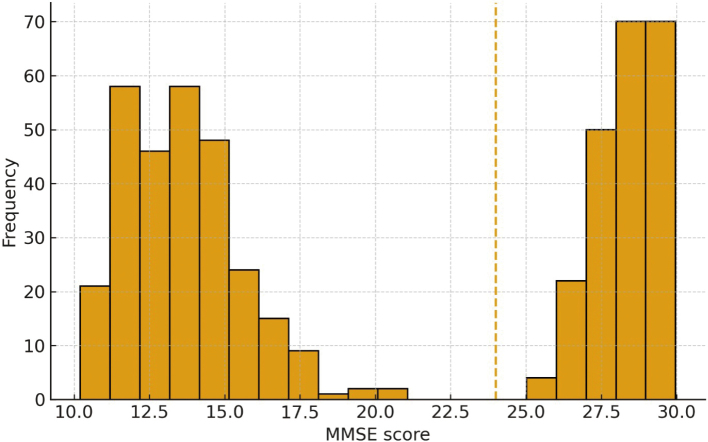
Distribution of Mini-Mental State Examination (MMSE) total scores (N = 500). Higher scores indicate better cognition; a cutoff of ≥ 24 denotes normal cognition.

The distribution of the MMSE scores of 500 participants with 10 to 30 scores is indicated in Figure 1. The distribution is bimodal, with one cluster specified in 12-16 and another in 27-30. Based on the typical cut-off of 24 and above normal cognition, 216 participants (43 per cent) were cognitively normal (met the cutoff), and 284 (57 per cent) did not reach the cutoff (cognitively impaired). This indicates that the sample had more cognitive impairment.

### Anxiety (HADS-A)

**Figure 2:**
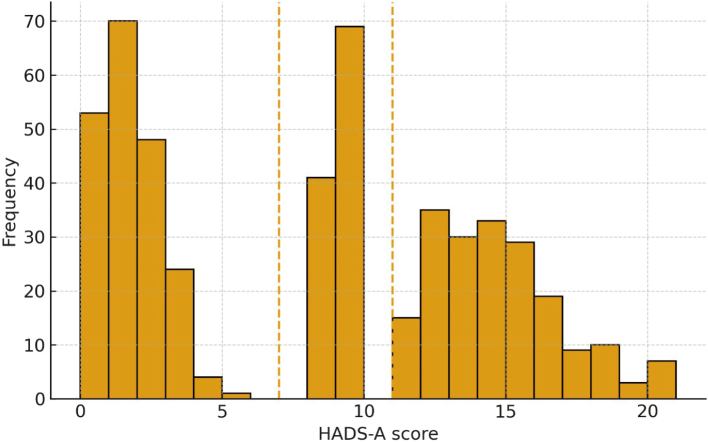
Distribution of Hospital Anxiety and Depression Scale-Anxiety (HADS-A) scores (N = 500). Cutoffs: expected (≤ 7), borderline (8-10), clinical (≥ 11)

Figure 2 shows the distribution of HADS-Anxiety scores among 500 subjects within a range of 0 to 21. The data indicate that there are three specific groups according to the predetermined cutoffs: normal (≤ 7), borderline (8-10), and clinical anxiety (≥ 11). In this sample, 200 (40%) were in the normal range, 110 (22%) in the borderline range, and 190 (38%) in the clinical range, which means that over half of the participants (60%) had levels of anxiety in the borderline to clinical range.

### Depression (HADS-D)

**Figure 3:**
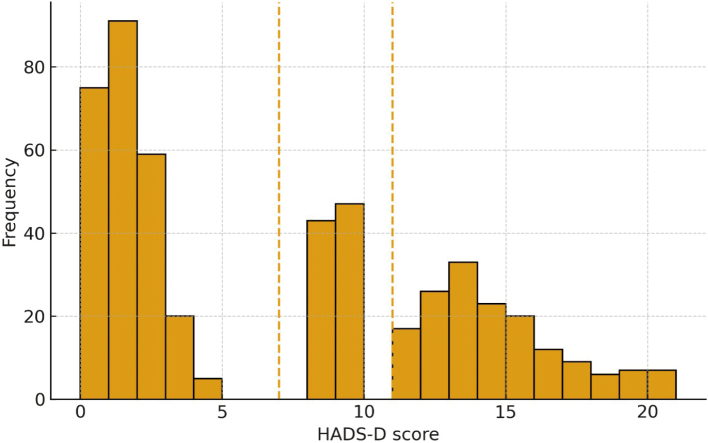
Distribution of Hospital Anxiety and Depression Scale-Depression (HADS-D) scores (N = 500). Cutoffs: expected (≤ 7), borderline (8-10), clinical (≥ 11)

Figure 3 presents the distribution of HADS- Depression scores in 500 participants, with minimum and maximum scores of 0 and 21, respectively. Using cutoff values, 500 participants were divided into two groups (250/500), 90 (18%) in the borderline range (8-10), and 160 (32%) in the clinical range (≥ 11). It means that approximately half of the sample reported at least borderline to clinical levels of depressive symptoms, and a third met the criteria of clinical depression.

### Quality of life (SF-36)

**Figure 4:**
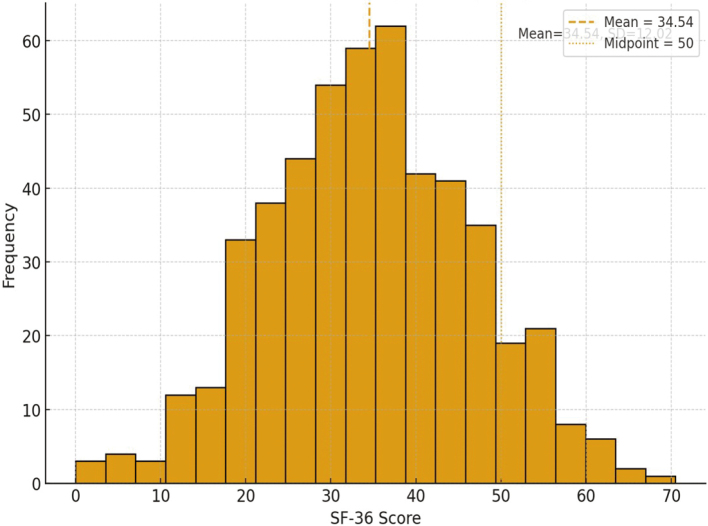
Distribution of Short Form-36 (SF-36) scores (N = 500). Higher scores reflect better health-related quality of life.

Figure 4 demonstrates the distribution of SF-36 quality of life scores across 500 participants, where most participants were located between 25 and 45, with a mean of around 35 (SD of 12). Because the SF-36 is a 0-100 scale, with higher scores showing better health-related quality of life, the presence of scores below the centre of 50 shows that the majority of the respondents had poor QOL overall in this research.

### Association of ICU length of stay with post-ICU outcomes

Table 2 demonstrates that there are significant relationships between post-ICU outcomes and ICU length of stay. A more extended ICU stay was moderately related to worse cognitive performance (MMSE: r = 0.25, p < 0.001) and lower quality of life (SF: r = 0.22, p < 0.001), and positively related to increased anxiety (HADS-A: r = 0.30, p < 0.001) and depression (HADS- D: r = 0.28, p < 0.001). The cognitive function (MMSE) had negative correlations with anxiety (r = -0.18, p < 0.001) and depression (r = -0.20, p < 0.001), as well as a positive correlation with quality of life (r = 0.24, p < 0.001). Depression and anxiety were highly correlated (r = 0.35, p < 0.001), and both were negatively correlated with quality of life (HADS-A: r = -0.26, p < 0.001; HADS-D: r = -0.29, p < 0.001).

**Table 2:** Spearman correlations between ICU length of stay and post-ICU outcomes (N = 500)

Variables	ICU length of stay	Total MMSE	Total HADS-A	Total HADS-D	Total SF-36
ICU length of stay	-	-0.25**	0.30**	0.28**	-0.22**
Total MMSE	-	-	-0.18**	-0.20**	0.24**
Total HADS-A	-	-	-	0.35**	-0.26**
Total HADS-D	-	-	-	-	-0.29**
Total SF-36 (QOL)	-	-	-	-	-

correlation = Spearman's; MMSE = Mini mental state examination; HADS-A = HADS = Hospital Anxiety and Depression Scale-Anxiety; HADS-D = HADS = Hospital Anxiety and Depression Scale-Depression; SF-36 = Short form questionnaire; p < 0.001 indicates statistical significance.

### Gender differences in post-ICU outcomes

Table 3 illustrates that there are significant gender differences in all post-ICU outcomes. Women experienced more cognitive impairment (low MMSE scores: U = 20,000, Z = -4.85, r = 0.22, p = 0.001) and anxiety (U = 21,600, Z = -4.25, r = 0.19, p = 0.001) and depression (U = 22,400, Z = -4.55, r = 0.20, p = 0.001) than men Conversely, men had higher quality of life (greater SF-36 scores: U = 18,500, Z = -5.10, r = 0.23, p < 0.001).

**Table 3:** Gender differences in cognitive, psychological, and quality of life outcomes after ICU (N = 500)

Outcome	Gender	N	Mean rank	Sum of ranks	Mann-Whitney U	Z	*p* (2-tailed)	*r*
Total MMSE (Cognition)	Male	320	210.00	67,200.00	20,000.0	-4.85	<0.001**	0.22
	Female	180	310.00	55,800.00	-	-	-	-
Total HADS-A (Anxiety)	Male	320	220.00	70,400.00	21,600.0	-4.25	<0.001**	0.19
	Female	180	300.00	54,000.00	-	-	-	-
Total HADS-D (Depression)	Male	320	215.00	68,800.00	22,400.0	-4.55	<0.001**	0.20
	Female	180	305.00	54,900.00	-	-	-	-
Total SF-36 (Quality of Life)	Male	320	310.00	99,200.00	18,500.0	-5.10	<0.001**	0.23
	Female	180	180.00	32,400.00	-	-	-	-

N = number of participants; MMSE Mini-Mental State Examination; HADS-A = HADS = Hospital Anxiety and Depression Scale-Anxiety; HADS-D= HADS = Hospital Anxiety and Depression Scale-Depression; SF-36 = Short form questionnaire; r is the effect size calculated as Z/√N, where N = 500; U = Mann-Whitney U tests; **p < 0.001 considered significant

### Age group differences in post-ICU outcomes

According to Table 4, there is a significant difference in all post-ICU outcomes considering age. Younger participants (18-29 years) had the highest mean ranks for cognition (MMSE). They reported lower anxiety, depression, and a better quality of life. In contrast, older adults, notably those aged 60 years and above, showed the most significant cognitive decline (χ^2^ = 42.0, p < 0.001, r = 0.29), higher psychological distress (HADS-A: χ^2^ = 28.5, p < 0.001, r = 0.24; HADS-D: χ² = 32.1, p < 0.001, r = 0.25), and poorer quality of life (SF-36: χ^2^ = 26.7, p < 0.001, r = 0.23).

**Table 4:** Age group differences in cognitive, psychological, and quality of life outcomes after ICU (N = 500)

Outcome	Age group	N	Mean rank
Total MMSE (Cognition)	18-29 years	50	310.00
	30-39 years	75	280.00
	40-49 years	100	250.00
	50-59 years	125	220.00
	60+ years	150	180.00
Total HADS-A (Anxiety)	18-29 years	50	310.00
	30-39 years	75	300.00
	40-49 years	100	280.00
	50-59 years	125	230.00
	60+ years	150	190.00
Total HADS-D (Depression)	18-29 years	50	305.00
	30-39 years	75	295.00
	40-49 years	100	270.00
	50-59 years	125	225.00
	60+ years	150	185.00
Total SF-36 (Quality of Life)	18-29 years	50	290.00
	30-39 years	75	280.00
	40-49 years	100	260.00
	50-59 years	125	230.00
	60+ years	150	200.00
Test statistics	*x*^2^(df = 4)	*p*	*r*
Total MMSE (Cognition)	42.0	<0.001**	0.29
Total HADS-A (Anxiety)	28.5	<0.001**	0.24
Total HADS-D (Depression)	32.1	<0.001**	0.25
Total SF-36 (Quality of Life)	26.7	<0.001**	0.23

N = number of participants; df = degree of freedom; MMSE = Mini mental state examination; HADS-A = HADS = Hospital Anxiety and Depression Scale-Anxiety; HADS-D = HADS = Hospital Anxiety and Depression Scale-Depression; SF-36 = Short form questionnaire; Results are from Kruskal-Wallis tests; *r* = effect size; **p < 0.001 consider significant

### Predictors of post-ICU outcomes

Table 5 indicates that the duration of stay in the ICU, age, gender, and the existence of comorbidities are essential factors in post-ICU outcomes. In particular, the longer the ICU stay, the worse the cognitive functioning of individuals (MMSE), the greater the anxiety (HADS-A) and depression (HADS-D), and the worse the quality of life (SF-36). Older age was associated with a decrease in cognitive function and quality of life, but also with a reduction in anxiety and depression. Women showed superior cognitive functioning but more anxiety and depression, as well as worse quality of life, than men. There was a consistent association of comorbidity with poorer outcomes in all domains. In general, ICU length of stay and comorbidity were found to be strong predictors of poor cognitive, psychological, and QOL outcomes in post-ICU patients' function.

**Table 5:** Multiple Regression Analyses Predicting Cognitive, Psychological, and Quality of Life Outcomes in Post-ICU Survivors (N = 500)

Predictor	B	SE	β	t	*p*	LL	UL
Constant (MMSE)	33.200	2.600	-	12.77	<0.001**	28.1	38.3
ICU length of stay	-0.900	0.240	-0.220	-3.75	<0.001**	-1.37	-0.43
Age	-0.340	0.120	-0.190	-2.83	0.005**	-0.58	-0.10
Gender (female = 1, male = 0)	2.100	0.700	0.150	3.00	0.003**	0.73	3.47
Comorbidities	-2.000	0.780	-0.140	-2.56	0.011*	-3.53	-0.47
Predictor	B	SE	Beta	t	*p*	LL	UL
Constant (HADS-A)	13.900	1.400	-	9.93	<0.001**	11.1	16.7
ICU length of stay	0.500	0.120	0.230	4.17	<0.001**	0.26	0.74
Age	-0.180	0.080	-0.120	-2.25	0.025*	-0.34	-0.02
Gender (female = 1, male = 0)	1.700	0.600	0.150	2.83	0.005**	0.52	2.88
Comorbidities	1.400	0.650	0.110	2.15	0.032*	0.12	2.68
Predictor	B	SE	Beta	t	*p*	LL	UL
Constant (HADS-D)	12.800	1.300	-	9.85	<0.001**	10.2	15.4
ICU length of stay	0.450	0.110	0.220	4.09	<0.001**	0.23	0.67
Age	-0.160	0.070	-0.110	-2.29	0.022*	-0.30	-0.02
Gender (female = 1, male = 0)	1.600	0.550	0.150	2.91	0.004**	0.52	2.68
Comorbidities	1.300	0.600	0.100	2.17	0.030*	0.13	2.47
Predictor	B	SE	Beta	t	*p*	LL	UL
Constant (SF-36)	96.200	3.000	-	32.07	<0.001**	90.3	102.1
ICU length of stay	-1.200	0.280	-0.200	-4.29	<0.001**	-1.74	-0.66
Age	-0.400	0.130	-0.150	-3.08	0.002**	-0.65	-0.15
Gender (female = 1, male = 0)	-3.800	1.200	-0.160	-3.17	0.002**	-6.17	-1.43
Comorbidities	-3.200	1.400	-0.120	-2.29	0.022*	-5.96	-0.44

Mini-Mental State Examination (MMSE; cognition), Hospital Anxiety and Depression Scale-Anxiety (HADS-A), Hospital Anxiety and Depression Scale-Depression (HADS-D), and SF-36 Quality of Life (SF-36); B = Unstandardized coefficients; SE = standard errors; β = standardized coefficients; t = test statistics; *p* = significance levels; Cl = confidence intervals; LL = lower limit; UL = upper limit; *p < 0.05, **p < 0.01, **p < 0.001.

### Association of age with cognitive impairment after ICU

Table 6 indicates that there is a significant relationship between age group and cognitive impairment, χ^2^ (4, N = 500) = 120.5, p < 0.001. The prevalence of cognitive impairment rose with age, being 20% of participants aged 18-29 years, 33% of those aged 30-39 years, 50% of those aged 40-49 years, 64% of those aged 50-59 years, and 79% of those aged 60 years and above.

**Table 6:** Association Between Age Group and Cognitive Impairment in Post-ICU Survivors (N = 500)

MMSE group	18-29 years; N(%)	30-39 years; N(%)	40-49 years; N(%)	50-59 years; N(%)	60+ years; N(%)	Total	*x*^2^(*df* = 4)	*p*
Normal	40 (80%)	50 (67%)	50 (50%)	45 (36%)	31 (21%)	216 (43%)	-	-
Impaired	10 (20%)	25 (33%)	50 (50%)	80 (64%)	119 (79%)	284 (57%)	-	-
Total	50 (100%)	75 (100%)	100 (100%)	125 (100%)	150 (100%)	500 (100%)	120.5	<0.001**

Frequency; % = percentage; df = degree of freedom; *x*^2^ = chi-square statistics; *p* = level of significance; *p*-values calculated using the chi-square test; MMSE = Mini-Mental State Examination; A significant chi-square test indicated that cognitive impairment increased with age, χ^2^(4, N = 500) = 120.5, **p < 0.001 consider significant.

### Association of gender with ICU length of stay

Table 7 indicates that there is a strong relationship between gender and length of stay in an ICU, χ^2^(3, N = 500) = 24.8, p < 0.001. The females were more likely to stay in the ICU longer than 7 days (57% vs. 34% of men), but the males tended to spend less time in the ICU (66% vs. 43% of females).

**Table 7:** Association between ICU length of stay and gender in post-ICU survivors (N = 500)

ICU length of stay	Male; N(%)	Female; N(%)	Total	*x*^2^(*df* = 3)	*p*
< 3 days	70 (22%)	17 (9%)	87 (17%)	-	-
3-6 days	140 (44%)	61 (34%)	201 (40%)	-	-
7-13 days	90 (28%)	74 (41%)	164 (33%)	-	-
≥ 14 days	20 (6%)	28 (16%)	48 (10%)	-	-
Total	320 (100%)	180 (100%)	500 (100%)	24.8	<0.001**

Frequency; % = percentage; df = degree of freedom; *x*^2^ = chi-square statistics; *p* = level of significance; *p*-values calculated using the chi-square test; Pearson’s chi-square test indicated a significant association between gender and ICU length of stay, χ^2^(3, N = 500) = 24.8, **p < 0.001 consider significant.

## Discussion

The research offers valuable evidence on neurocognitive, psychological, and quality-of-life outcomes of patients who survived sepsis in Pakistan after ICU discharge. In our study, poor cognitive performance was linked to a more extended ICU stay, which is in line with the results of prior reviews that suggested that ICU survivors are more prone to the development of long-term cognitive impairment^14)^. Also, we found that a more extended ICU stay was related to increased anxiety and depressive symptoms, which is aligned with the literature indicating that psychological distress has a high burden among ICU survivors. Whereas previous studies have noted that post-discharge care requirements and living situations are key risk factors, our findings indicate that the ICU course in itself, especially length of stay, is equally critical in influencing these outcomes^15)^.

Prolonged ICU stay was also a predictor of worse QOL, in agreement with prior studies that found that prolonged hospitalisation and severity of illness led to long-term decreases in the quality of life among ICU survivors^16)^. Our result that poorer cognition was linked to higher anxiety and depression is in line with the earlier findings that demonstrate that psychological symptoms intensify with cognitive impairment. Other studies, however, indicate that these symptoms can attenuate in severe forms, probably because of loss of insight, something that our data has not recorded^17)^.

Poor cognition was also linked to worse quality of life, which is in favour of the idea that both subjective and objective cognitive deficits provoke functional and psychosocial restrictions in everyday life^18)^. The correlation between anxiety and depression was found to have a significant positive relationship, which is in line with the tripartite model of the subject matter, where the symptoms of distress overlap in anxiety and depression conditions^19)^. Furthermore, the increased psychological symptoms are linked to a poorer quality of life, which is similar to the longitudinal evidence regarding the decrease in QOL during periods of anxiety and depression and persistence of lower levels of QOL following symptom disappearance^20)^.

In addition to these general associations, we also found significant differences based on gender in our study. The fact that females reported greater MMSE scores is consistent with previous data indicating that females are also comparatively stronger in verbal memory, fluency, and learning tasks. It is also observed in literature that sex hormones can also affect cognitive functions, which might be a partial explanation of the observed gender differences in post-ICU cognition^21)^. Our results indicate that females had more anxiety and depression symptoms, and worse QOL, which is aligned with the population-based data indicating that women are at increased risk of affective disorders, and frequently report worse overall quality of life than men^22, 23)^.

Age also proved to be another critical post-ICU outcome determinant. Our findings demonstrate that cognitive performance decreases with age during the post-ICU period, which aligns with the research on cognitive ageing that suggests the decrease of process-based cognitive abilities like memory and reasoning^24)^. Likewise, older ICU survivors experienced poorer quality of life, which is consistent with the evidence that age-related factors lead to long-term decreases in QOL in older adults^25)^. The observation that younger survivors of the ICU experienced more anxiety and depressive symptoms than older participants is in line with the findings that indicate an age-related reduction in vulnerability to affective symptoms in adulthood^26)^.

We found that increased comorbidity burden was correlated with poorer cognition, more anxiety and depression, and reduced QOL in post-ICU patients, which aligns with previous studies that indicate that the cumulative effect of chronic conditions on overall well-being^27-29)^. We further found that there was a distinct age gradient, with the rate of cognitive impairment rising significantly with older age groups, which supports the importance of age as a key factor in the post-ICU cognitive decline^24)^.

Lastly, in addition to the cognitive, psychological, and comorbidity factors, our results also revealed gender-specific differences in ICU stay patterns. In our findings, female ICU patients were more likely to have prolonged ICU hospitalisation than men. Other gender differences in ICU stay have also been identified in surgical groups, with women having more extended ICU and postoperative stay than men^30)^. Even though our patients were general ICU survivors, not surgical, these results indicate that the female patients might be more susceptible to prolonged critical care needs.

This research has a number of limitations that must be taken into consideration in the interpretation of the findings. The cross-sectional design limits the possibility of inferences regarding causality and fails to intercept the pattern of recovery over time. The generalizability is also restricted by convenience sampling in tertiary hospitals in Islamabad because these sample types do not represent the rural population or the lower-tier healthcare facilities. Moreover, even though validated instruments were employed, including MMSE, HADS, and SF-36, the self-reported questionnaire also creates the risk of reporting bias and can miss subtle neurocognitive impairment that cannot be identified by a test-retest. It is also hard to differentiate between new and pre-existing impairments due to the lack of baseline pre-ICU cognitive and psychological assessment. Also, all other unmeasured variables, including sedation practices, mechanical ventilation time, and post-discharge rehabilitation availability, could have affected the outcomes but were not measured.

Future studies must employ longitudinal cohort designs to track cognitive, psychological, and QOL outcomes in the long term, preferably up to 12 months or beyond after ICU discharge. Research that includes objective neuropsychological measures and neuroinflammatory biomarkers would enhance the knowledge of the processes that underlie post- ICU syndrome. Interventional studies are also required to assess the effectiveness of the structured post-ICU rehabilitation program, such as cognitive training, physical therapy, and psychological counselling, especially in health care systems with limited resources. Also, further research ought to examine the effect of cultural and socioeconomic influences on the recovery and methods to incorporate family and community systems of support within the survivorship care.

## Conclusion

Overall, the research indicates that survivors of sepsis in Pakistan experience a high rate of cognitive impairment, psychological distress and low QOL within weeks of leaving the ICU. Predictors of poor outcomes were longer ICU stay, older age, female sex, and comorbidities. The results highlight the necessity of systematic follow-up care after ICU, early detection of neurocognitive and psychological complications, and specific rehabilitation interventions for vulnerable groups. Overcoming these challenges, healthcare systems will be able to increase the number of survivors and contribute to the long-term survivability of survivors of sepsis.

## Author contributions

AKVK conceived and designed the study and supervised the overall project. MI contributed to the study design, psychological assessments, and interpretation of HADS outcomes. IAS assisted in data acquisition and clinical interpretation. HJ contributed to patient recruitment and collection of hospital data, while AH supported data collection and clinical analysis. NA was responsible for statistical analysis and drafting of results. MN participated in the literature review and manuscript drafting. AI assisted in data entry and quality checks. MWA contributed to the study methodology and revision of the manuscript. SHK assisted with data management and formatting of tables and figures. ZS drafted the manuscript, coordinated revisions, and served as the corresponding author. SB contributed to the literature review, proofreading, and critical revision of the manuscript. All authors read and approved the final manuscript.

## Conflicts of interest statement

The authors declare that there are no conflicts of interest.
